# Sodium and Potassium Content of the Most Commonly Available Street Foods in Maputo, Mozambique

**DOI:** 10.3390/foods11050688

**Published:** 2022-02-25

**Authors:** Neusa Jessen, Sofia Sousa, Marcello Gelormini, Susana Casal, Olívia Pinho, Pedro Moreira, Albertino Damasceno, Patrícia Padrão, Nuno Lunet

**Affiliations:** 1 Epidemiology Research Unit (EPIUnit), Instituto de Saúde Pública, Universidade do Porto, 4050-600 Porto, Portugal; neusa.jessen@gmail.com (N.J.); sofiaazevedodesousa@gmail.com (S.S.); sucasal@ff.up.pt (S.C.); pedromoreira@fcna.up.pt (P.M.); tino_7117@yahoo.com.br (A.D.); padraopatricia@gmail.com (P.P.); 2 Faculdade de Medicina, Departamento de Medicina Interna, Universidade Eduardo Mondlane, Maputo 1100, Mozambique; 3 Faculdade de Ciências da Nutrição e Alimentação, Universidade do Porto, 4150-180 Porto, Portugal; oliviapinho@fcna.up.pt; 4 Agência Italiana para a Cooperação e Desenvolvimento, Maputo 1100, Mozambique; marcello.gelormini@gmail.com; 5 Requimte, Laboratório de Bromatologia e Hidrologia, Faculdade de Farmácia, Universidade do Porto, 4050-313 Porto, Portugal; 6 Departamento de Ciências da Saúde Pública e Forenses e Educação Médica, Faculdade de Medicina, Universidade do Porto, Alameda Prof. Hernâni Monteiro, 4200-319 Porto, Portugal

**Keywords:** sodium, potassium, street food, Mozambique

## Abstract

Street foods can contribute largely for dietary sodium intake of populations in developing countries. We aimed to assess the variability in sodium and potassium composition of the most commonly available homemade street foods in Maputo city, capital of Mozambique. In a cross-sectional evaluation, researchers canvassed areas with 500-m diameter centered around 20 randomly selected public transport stops, identified all street food vending sites and, in randomly selected sites, purchased 56 samples of the most frequently available homemade foods. Samples were analyzed for sodium and potassium concentrations, using flame photometry. The 56 samples represented main dishes (45 samples of 12 types of food item), sandwiches (8 samples of 5 types of food item) and fried snacks (3 samples of 2 types of food item). Median contents (range), in mg/serving, were 921 (198 to 2525) of sodium and 385 (24 to 1140) of potassium. Median (range) of sodium to potassium molar ratio was 4.1 (1.3 to 41.5). One serving of main dishes was estimated to contribute from 32.1% to 99.9% of the recommended maximum daily sodium intake. The present study shows a large variability and potential for improvement in sodium and potassium contents of homemade foods frequently available in the streets of Maputo city.

## 1. Introduction

Non-communicable diseases (NCD), the leading causes of mortality worldwide, present greater and increasing importance in low- and middle-income countries (LMIC) [1]. Among NCD, Cardiovascular diseases (CVD) represent the first cause of deaths [1], remaining the leading cause of morbidity and mortality around the world [2]. High systolic blood pressure (SBP), the main risk factor for CVD, was estimated to be responsible for 10.8 million deaths in 2019, remaining positioned as the major global risk factor for preventable death [3]. The close relation between high sodium (Na) and low potassium (K) diets and the development of high blood pressure has been widely demonstrated, along with the effect of dietary salt reduction in the control of hypertension [4,5,6,7].

The daily intake recommendations of the World Health Organization (WHO) comprise a maximum of 2000 mg of Na (corresponding to five grams of salt) [8] and a minimum of 3510 mg of K [9]. In addition, the molar ratio of Na to K (Na:K ratio) should be kept at one [10]. However, in the majority of the studied populations around the world, Na intakes were found to be well above the WHO recommendations [11], including in most of the sub-Saharan Africa countries [12], and K intakes are usually unsatisfactory [13]. Specifically in Mozambique, the prevalence of hypertension is high and increasing (25–64 years: 38.9% in 2015, vs. 33.1% in 2005) [14,15], and the only study that evaluated Na and K urinary excretion in a sample of Maputo inhabitants showed that more than 90% of the participants did not meet the WHO recommendations [16].

The term “street food” was defined in 1989 by the Food and Agriculture Organization of the United Nations [17], and, restated by the WHO in 1996 [18], as “ready-to-eat foods and beverages prepared and/or sold by vendors and handlers especially in streets and other similar public places, for immediate consumption or consumption at a later stage without further processing or preparation”. Street foods, which are usually cheap, culturally adapted and broadly distributed, may vary in Na and K content and may represent an important source of Na in the diet of urban populations of countries under rapid urbanization and nutritional transition, such as Mozambique [19,20]. Thus, we aimed to assess the variability in the Na and K composition of homemade street foods most commonly available in urban Maputo, Mozambique.

## 2. Materials and Methods

A cross-sectional evaluation of a sample of street food vending sites was conducted between October and December 2014, in the district of KaMpfumu, the most urbanized among the seven municipal districts of the administrative repartition of the city of Maputo [21], capital of Mozambique. This study is part of a project aiming to characterize street food offer and to assess the nutritional composition of the most commonly available homemade foods in the city of Maputo, as previously described in detail [22].

### 2.1. Sampling Procedures

The distribution of public transport stops within the district of KaMpfumu, according to the Government of Mozambique [23] and other maps produced by the Maputo Municipal Council (Conselho Municipal Cidade de Maputo, Maputo, Mozambique) [24], was used for the sampling procedure. A total of 134 public transport stops were identified and 20 were randomly selected. The study area corresponded to the 500-m buffers around each selected stop, excluding those portions that fall outside the administrative borders of the municipal district and overlapping areas. Researchers, working in pairs, canvassed the study area to identify all street food vending sites and characterize their street food offer. A total of 968 street food vending sites were identified, and 810 (83.7%) agreed to be interviewed. As part of the project [22], some information was obtained, namely regarding gender of vendors, characteristics of the business, type of food products available, date and place of preparation and size and price of the portions of homemade foods. The food products sold were identified and ready-to-eat food products were classified as homemade or industrial. For the purpose of this study, homemade foods were defined as foods that were cooked and/or prepared at home or on the street, even if using industrial ingredients. Samples (each sample corresponding to a portion being sold) of the most frequently available food products identified as homemade were purchased in randomly selected vending sites and analyzed.

### 2.2. Food Sample Collection and Bromatological Analysis

A sample of each food product (corresponding to one portion) was bought from different vending sites, comprising a total of 56 homemade street food samples. The samples were individually packed in small containers and weighed before being properly stored in freezer at −18 °C, until bromatological analysis was conducted.

Before analysis, samples were defrosted and total weight was compared to detect moisture losses during storage and shipping. Concentrations of Na and K were evaluated by flame photometry [25]. Analyses were conducted in duplicate. Proximate analysis was performed in accordance with the standard Association of The Official Analytical Chemists (AOAC, Rockville, MD, USA) International methods [26]. The caloric value of samples was estimated after proximate analysis of the food components has been carried out, including: moisture analysis (oven-drying at 103 °C until constant weight), total fat determination (Soxhlet procedure), protein content determination (Kjeldahl procedure), total carbohydrates plus fibre estimation (by difference) [26]. Results were expressed in milligrams (mg)/serving and mg/2000 kilocalories (kcal).

### 2.3. Data Analysis

**Figure 1:**
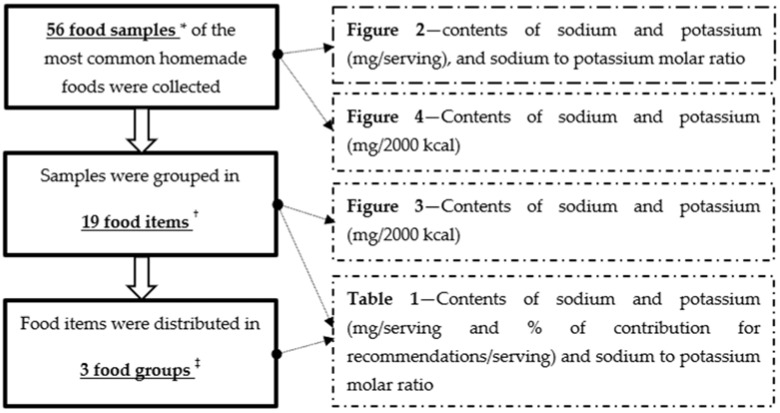
Flow chart of the data analysis process. * Two aliquots from each sample were analysed (bromatological analysis). The mean of the two values was used for data analyses considering each of the 56 samples. ^†^ The mean of the values obtained from the individual samples of each food item was computed. ^‡^ The mean of the values obtained from the individual samples of foods pertaining to each food group was computed.

Figure 1 describes the data analysis process.

The 56 food samples corresponded to 19 different food items, which were further grouped, according to the WHO nutrient profile model [27], in: (i) main dishes, (ii) sandwiches and (iii) fried snacks.

For individual food samples, the average of two measurements was used to calculate Na and K contents, as mg/serving. To calculate individual molar Na:K ratios, contents of Na and K of each sample were converted into millimoles using their molar weights, 23.0 g/mol and 39.1 g/mol, respectively.

For each food item, the average serving sizes, in grams, contents of Na and K, in mg/serving, and molar Na:K ratio, were calculated as the mean of the values obtained from each individual sample. The percentage contributions of a serving of each food item to the maximum Na daily intake and minimum K daily intake, as recommended by WHO [8,18], were also computed.

Na and K contents of individual food samples and food items were also calculated as mg/2000 kcal, according to the reference daily intake for an average adult.

Since the study was an exploratory analysis to complement data on composition of homemade street foods, no statistical analysis requiring a specific sample size was defined in advance. Descriptive statistics were computed and used to present the results, Median and range was used to describe Na and K contents of all food samples collected. Mean and standard deviation/range were used to describe Na and K contents of the food items and the food groups. Box plots were produced to describe the distribution of Na, K and Na:K ratio of all food samples collected. Na and K contents, in mg/2000 kcal, for each of the 19 food items and 56 individual samples, were presented using scatter plots. Statistical analyses were performed using Stata^®^ version 15.0 (StataCorp., College Station, TX, USA).

## 3. Results

**Figure 2:**
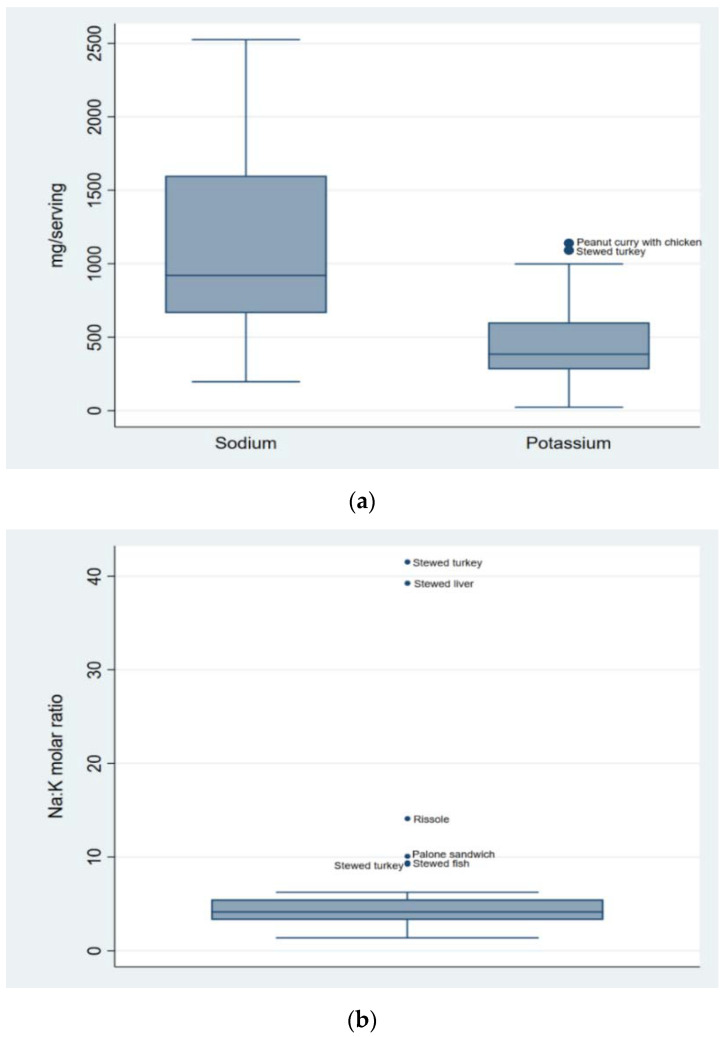
Contents of sodium and potassium (mg/serving) (**a**), and sodium to potassium molar ratio (**b**) of all food samples collected (*n* = 56). Palone, known as Polony in South Africa, is a sausage derived from mortadella. It can be made of beef, chicken, turkey, a combination or soy protein, plus condiments and fat. It is usually served cold, in slices. Rissole is a deep-fried pastry filled with a sauce of shrimp.

Figure 2 depicts the distribution of the Na and K contents (mg/serving) (Figure 2a) and the corresponding Na:K molar ratios (Figure 2b) in all food samples. The median Na content was 921 mg/serving, ranging from 198 to 2525 mg/serving, whereas the median K content was 385 mg/serving, ranging from 24 to 1140 mg/serving. A total of 42.9% of the samples exceeded 1000 mg of Na per serving, and 8.9% exceeded 2000 mg of Na per serving. The median (range) Na:K molar ratio was 4.1 (1.3 to 41.5).

**Figure 3:**
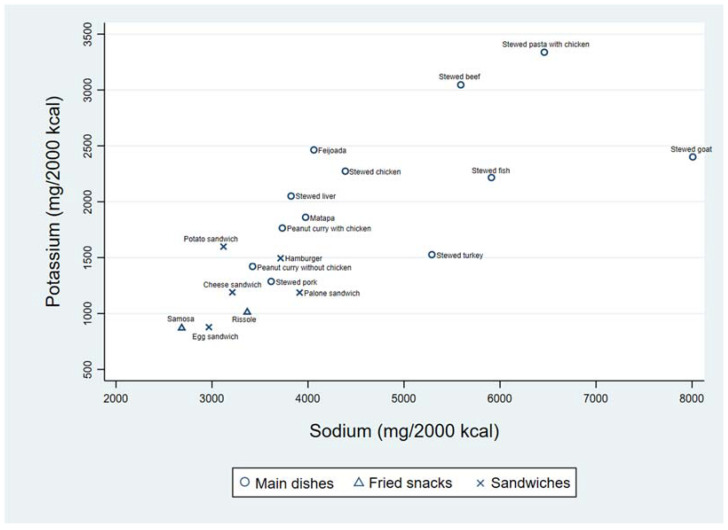
Mean contents of sodium and potassium (mg/2000 kcal) for each of the defined food items (*n* = 19). Feijoada is a traditional main dish which consists of Stewed beans with beef and/or pork; Matapa is a traditional main dish made with peanuts, coconut, cassava leaves and shrimps. Samosa is a crispy fried triangular pastry with a savoury filling that may be made with different ingredients such as minced meat, spiced chicken, vegetables or fish with onions, garlic and/or other spices.

Figure 3 presents the distribution of mean levels of Na and K per 2000 kcal, among each of the 19 food items. A wide variability in mean contents of Na and K was observed across the food items. The highest content of Na was observed in stewed goat (8008 mg/2000 kcal). The lowest content of Na was observed in a fried snack, samosa, (2685 mg/2000 kcal) which also fell short of K (870 mg/2000 kcal). Stewed pasta with chicken presented the highest K content, close to 3500 mg/2000 kcal, but it also presented a very high level of Na (6462 mg/2000 kcal).

**Figure 4:**
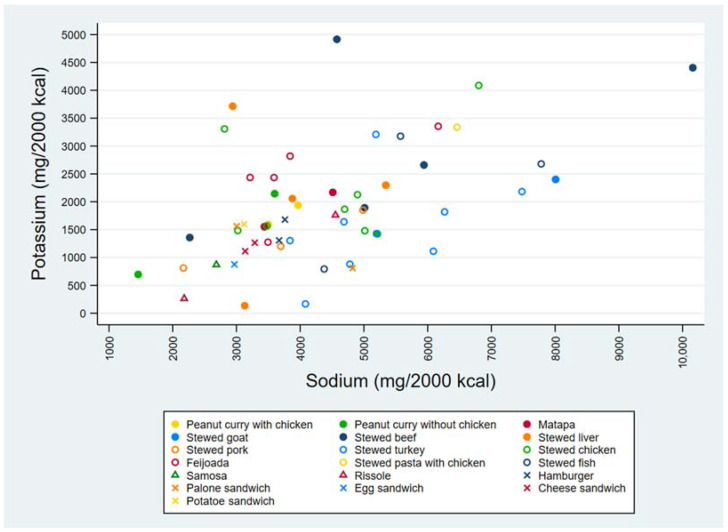
Contents of sodium and potassium (mg/2000 kcal) of all food samples collected (*n* = 56), distributed in three food groups: main dishes (●**○**), fried snacks (∆) and sandwiches (x).

Figure 4 displays the individual contents of Na and K of all collected food samples according to their corresponding food groups, in mg/2000 kcal. A large variability in Na and K contents can also be observed among the food samples, with most samples presenting more than 2000 mg Na/2000 kcal and less than 3500 mg K/2000 kcal. The highest Na content, exceeding 10,000 mg Na /2000 kcal, was observed in a sample of stewed beef. The lowest K content was found in samples of stewed liver, stewed turkey and rissoles, again with variations between different samples of similar foods.

Table 1 depicts the mean contents of Na and K, Na:K molar ratios per food item and per broad food group, as well as the percentage contribution of each food item to the daily Na and K recommendations. Main dishes were the main sources of both Na and K, with one serving contributing to almost two thirds of the maximum daily Na recommendation and to one quarter of the recommended minimum daily K intake. The highest Na values, among the main dishes, were observed in peanut curry with chicken, stewed turkey and stewed fish, followed by peanut curry without chicken and matapa. A serving of peanut curry with chicken contributed 99.9% to the recommended maximum daily Na intake. The levels of Na were also high in sandwiches. One serving of hamburgers contributed to just over 50% of the recommended maximum daily Na intake. Fried snacks, besides presenting a high content of Na, were shown to be poor in K, with an estimated molar Na:K ratio of 7.9.

All food items presented a mean molar Na:K ratio above the recommendations, ranging from 3.0 to 11.9.

## 4. Discussion

In the present study, the level of Na per serving was very high in most of the analysed food samples, with some food items contributing to a large proportion of the maximum daily recommendation of this nutrient. However, a wide variability was observed in Na contents across the samples.

When assessing the adequacy of Na in the diet, K levels and, particularly the Na to K relation should be considered. K attenuates the negative effects of Na [28,29], with even greater benefits for those with high Na intake [30], and the Na:K ratio is considered a stronger marker of the relation of Na to blood pressure and a better predictor of the outcomes of blood pressure [31,32,33,34]. The present study also shows a variable content of K amongst the food samples, with some of them presenting very low levels. In all of the food items, the Na:K ratio was found to be above one, the level recommended by the WHO [9,10]. These results are in accordance with previous studies conducted in the city of Maputo, showing high Na intake with most of the participants not meeting the recommended daily consumption of Na and K [16]. In the present study, when analyzing specific food items, it is notable that the most commonly found homemade foods in this environment are stews and curry which are usually cooked with vegetable oil, particularly coconut oil, which is mainly constituted of saturated fat (with around 120 calories in a tablespoon) is widely used in this setting. In addition, during culinary preparations, apart from adding salt, salty condiments, particularly powdered chicken stock, are frequently used as seasoning [16,35,36]. As such, just by ingesting one serving of a main dish, the individual will be taking a highly caloric meal that already provides most, if not all, of the recommended daily amount of salt. As an example, someone eating a meal of stewed goat, with the daily recommended calories for an adult (2000 kcal), will be consuming four times the maximum daily recommendation of Na. Even the food item that presented the lowest content of Na, a snack called samosa, if 2000 kcal are consumed, will provide over the maximum daily recommendation of Na. Sandwiches, though varying according to the filling, also provide very high amounts of Na. In developing countries, street foods already represent an important contributor to the daily diet of many populations [20]. In Mozambique, undergoing a process of rapid urbanization [37], the demand and the offer of street foods and, hence, its contribution to the daily diet of the population is expected to grow. In fact, the offer of street food is abundant in Maputo (hundreds of vending sites were found in one district) and the type of foods being sold also reflect the ongoing nutrition transition in this setting, with coexistence of natural foods and homemade dishes with highly processed industrialized food products. In addition, the last national Family Budget Survey revealed that the proportion of the total household food expenses allocated to meals consumed outside home increased from 2008/09 to 2014/15, particularly in urban centers and in the south region of the country (urban south: from 1.7 to 28.3) [38]. In accordance, the prevalence of overweight and obesity in adults increased significantly from 2005 to 2014/15 (women: 18.3 to 30.5%, *p* < 0.001; men: from 11.7 to 18.2%, *p* < 0.001) [39], another sign of the ongoing nutritional transition, with adoption of energy dense and fat rich foods, particularly in urban centers.

People with medium living standards may require particular attention, such as found in a national survey performed in South Africa, where this fraction of the population was shown to consume street foods more frequently [40]. Yet, studies evaluating the micronutrients in the composition of these foods are scarce [41]. Among LMIC, a similar evaluation was conducted in Tajikistan and Kyrgyzstan and, in line with our observations in the city of Maputo, the authors found highly variable contents of Na and K in different foods and in samples of similar foods collected from different vendors [42].

Dietary interventions to reduce salt consumption at a population level have been shown to be cost effective and, as so, were selected by the WHO as one of the ‘best buys’ to prevent NCD [43]. Considering that the taste of salt can be altered when dietary salt is reduced over time, leading to a change in the preference to less salty foods [44,45], street food vendors and consumers should be targets of health and nutrition education programs. This is of special importance in Mozambique, where it has been shown that there is a general knowledge of the population regarding the prejudicial health effects of high salt diets but yet, perception of high salt intake is very low and most of the population admitted not adopting any of the behaviours known to reduce salt consumption [35]. Furthermore, in a sample of Maputo inhabitants, the most important source of daily salt intake was found to be discretionary salt [16]. Since hypertension represents a great health problem in the country [15], the high Na content in street foods may play a role in the already growing burden of NCD [46]. Even so, it should be considered that street foods may represent the solely source of income for some people, especially for women in LMIC, where it is largely unregulated and do not require high investment [20] and this situation is likely to persist while the needs and services of the urban poor are disregarded and unprotected. In fact, in the city of Maputo, more women were found selling in stationary vending sites, which is in line with findings from other countries [47]. In fact, several aspects of the street food vending activity, such as preparation and even sale are usually the domain of women. However, a deeper understanding of how the local street food trade market operates is warranted to raise important insights, such as the drivers for involvement in that market, if there are rules governing it and the revenues obtained from it. This information would be important to guide interventions aimed to maximize the benefits of this economic activity for consumers, vendors and possible for the economy of the country. The street foods business may generate a good volume of sales and a high level of employment and has the nutritional potential [47]. Having in account evidence showing that in Mozambique [35] such as in other countries [48,49], women tend to present an overall higher knowledge and better behaviors regarding salt intake, the predominance of women in street food market should be seen as an advantage. Since in the African context women are usually the ones who acquire and prepare the food for the family and also, are more prone to follow health related counselling [50], they could assist in the implementation of health promotion and education activities at the community level. The government should shift the perception of this economic activity as a problem and formulate nutrition and hygiene programs, policies and regulations (including fair licensing and inspections) to improve the knowledge of the vendors and to protect both vendors and consumers. Of notice, salty seasonings should not be disregarded when designing strategies aimed to reduce population salt intake. The street food vendors should be encouraged to reformulate their homemade foods, to reduce the Na and increase K contents, in order to contribute for the establishment of a healthier food environment. The role of both men and women should be recognized and programmes developed to help them to become self-sufficient, especially those that have street food as their sole source of income. Women in particular, may encounter more barriers, including those related to childcare and sexual harassment, and should be protected, to avoid their displacement once the business improves. In fact, although food processing by cooking is frequently seen as women´s role and often perceived as of low economic value, markets are dynamic and may shift, in particular if upgrades are made and result in higher returns, the business may be overtaken by men.

It is also notable that although the number of papers addressing street food related topics increased exponentially in the last decade, several issues related to availability and consumption of street foods are yet poorly explored [41]. Despite the samples were collected in 2014 and street food offer may have changed in the last years, to the best of our knowledge, this is the first study providing this type of evidence from Mozambique and provides a baseline for further monitoring of the street food environment in Maputo. Future evaluations of street foods should also include the assessment of patterns of purchase and consumption by the population and selling volumes, which would be useful to estimate the relevance of these foods on the burden of CVD in this setting.

The present study provides data on Na and K contents of the homemade foods more frequently sold in the streets of the most urbanized province of Mozambique. Although these results cannot be generalized to other settings, such as rural areas, where the preparation of food may be different, they warrant attention from health advocates and authorities. The large variability in the levels of these nutrients across the analyzed food samples present a window of opportunity for action, with potential to reduce Na and increase K intake by the population and lead to subsequent health gains.

## Figures and Tables

**Table 1:** Mean contents of sodium and potassium and sodium to potassium molar ratio of the collected homemade foods (*n* = 56), within each food group.

Foods	n*	Mean Serving Size (g)	Sodium				Potassium				Molar Na:K Ratio	
			Mean mg/Serving (SD)	(Min–Max)	% Recom. ^†^	(Min–Max)	Mean mg/Serving (SD)	(Min–Max)	% Recom. ^‡^	(Min–Max)	Mean (SD)	(Min–Max)
Main dishes	45	351.5	1195 (607)	(380–2525)	59.7	(19.0–126.3)	497 (261)	(24–1140)	24.8	(1.2–57.0)	5.8 (7.8)	(1.3–41.5)
Peanut curry with chicken	2	657.5	1999 (335)	(1762–2235)	99.9	(88.1–111.8)	947 (205)	(802–1091)	47.3	(40.1–54.6)	3.6 (0.2)	(3.5–3.7)
Peanut curry without chicken	3	657.4	1359 (754)	(717–2189)	67.9	(35.8–109.5)	546 (183)	(342–697)	27.3	(17.1–34.8)	4.2 (1.8)	(2.9–6.2)
*Matapa*	2	437.5	1288 (96)	(1221–1356)	64.4	(61.0–67.8)	602 (71)	(551–652)	30.1	(27.6–32.6)	3.7 (0.2)	(3.5–3.8)
Stewed beef	5	220.9	661 (185)	(380–864)	33.0	(19.0–43.2)	369 (162)	(227–640)	18.5	(11.4–32.0)	3.3 (1.1)	(1.6–4.5)
Stewed pork	3	220.8	735 (191)	(543–925)	36.8	(27.2–46.3)	259 (50)	(203–300)	13.0	(10.2–15.0)	4.8 (0.4)	(4.5–5.2)
Stewed goat	1	270.9	1563		78.1		469		23.4		5.7	
Stewed turkey	9	479.2	1876 (439)	(1088–2525)	93.8	(54.4–126.3)	537 (310)	(61–1140)	26.8	(3.4–57.0)	10.1 (12.0)	(2.8–41.5)
Stewed chicken	7	270.8	950 (322)	(710–1636)	47.5	(35.5–81.8)	511 (279)	(271–983)	25.5	(13.5–49.2)	3.6 (1.3)	(1.4–5.8)
Stewed pasta with chicken	1	211.7	1090		54.5		563		28.2		3.3	
*Feijoada*	5	228.5	643 (125)	(523–807)	32.1	(26.2–40.3)	389 (100)	(229–496)	19.5	(11.4–24.8)	3.0 (1.0)	(2.2–4.7)
Stewed liver	4	217.3	707 (183)	(552–961)	35.4	(27.6–48.0)	393 (298)	(24–752)	19.6	(1.2–37.6)	11.9 (18.2)	(1.3–39.3)
Stewed fish	3	394.7	1789 (589)	(1220–2396)	89.5	(61.9–120.0)	681 (408)	(221–999)	34.1	(11.1–49.9)	5.8 (3.3)	(3.0–9.4)
Sandwiches	8	184.0	832 (227)	(596–1106)	41.6	(29.8–55.3)	307 (104)	(177–494)	15.3	(8.8–24.7)	5.0 (2.2)	(3.3–10.1)
Hamburger	2	241.0	1092 (17)	(1080–1104)	54.6	(54.0–55.2)	440 (77)	(386–494)	22.0	(19.3–24.7)	4.3 (0.7)	(3.8–4.8)
*Palone* sandwich	2	165.0	865 (341)	(624–1106)	43.2	(31.2–55.3)	256 (98)	(186–325)	12.8	(9.3–16.3)	6.7 (4.8)	(3.3–10.1)
Cheese sandwich	2	165.0	760 (39)	(733–787)	38.0	(36.6–39.4)	282 (30)	(261–304)	14.1	(13.0–15.2)	4.6 (0.3)	(4.4–4.8)
Egg sandwich	1	165.0	596		29.8		177		8.8		5.7	
Potatoe sandwich	1	165.0	630		31.5		323		16.2		3.3	
Fried snacks	3	58.1	236 (50)	(198–292)	11.8	(9.9–14.6)	68 (38)	(24–95)	3.4	(1.2–4.7)	7.9 (5.4)	(4.4–14.1)
Rissole	2	58.1	208 (14)	(198–218)	10.4	(9.9–10.9)	54 (43)	(24–84)	2.7	(1.2–4.2)	9.3 (6.9)	(4.4–14.1)
Samosa	1	58.1	292		14.6		95		4.7		5.2	

n* values correspond to the number of individual samples included in each food item. ^†^ WHO recommends sodium intake of less than 2000 mg/day [8]. ^‡^ WHO recommends potassium intake of at least 3510 mg/day [18].
